# Search Behavior in Goat (*Capra hircus*) Kids From Mothers Kept at Different Animal Densities Throughout Pregnancy

**DOI:** 10.3389/fvets.2019.00021

**Published:** 2019-02-11

**Authors:** Judit Vas, Rachel M. Chojnacki, Inger Lise Andersen

**Affiliations:** Department of Animal and Aquacultural Sciences, Faculty of Biosciences, Norwegian University of Life Sciences, Ås, Norway

**Keywords:** search behavior, goat, cognition, prenatal effect, sex, object permanence

## Abstract

Individual differences in cognitive performance are often reported but factors related to variation within species are rarely addressed. Goats *(Capra hircus)* have been subjects of many cognitive studies recently but without focus on individual variation. Among others, factors such as prenatal stress and sex of the individual have been proposed as possible explanations for individual variation in cognitive skills. We aimed to study whether prenatal environment, prenatal stress, litter size, sex, and birth weight influences search behavior skills of goat kids. Pregnant Norwegian dairy goats were exposed to different spatial allowance (namely 1.0, 2.0, or 3.0 m^2^ per animal) within the commercially applied range during pregnancy and their serum cortisol levels were measured six times within this period. Twenty-six of the kids born entered a three-stage searching task with increasing difficulty when they were 6 weeks old. The tasks included finding a bucket of milk: while moving (stage 1), after moving and disappearing behind a curtain (stage 2), and moving behind a displacement device and the device moving behind a curtain while hiding the bucket (stage 3). We found that prenatal animal density had no effect on the search skills of the offspring, while kids with higher prenatal maternal cortisol levels performed better at the highest stage tested: finding an object after single invisible displacement. At this stage, singleton kids and males performed better than twins and females. Birth weight had no effect at this stage. The findings suggest that maternal cortisol in the observed range had a facilitating effect on cognitive development of goat kids.

## Introduction

Animals often follow the trajectories of prey, predators, and conspecifics; however, should the object become hidden, an animal which has the ability to mentally reconstruct the object would have a distinct advantage (1, 2). Searching behavior, observed when animals make attempts to find objects, may be a manifestation of object permanence skills (3). Object permanence is the cognitive capacity to understand that objects continue to exist even when they have disappeared from view and the ability to represent their unseen displacement trajectory. Standardized tests based on Piaget's theory of object permanence (3) are widely used in developmental and comparative research [reviewed by Jaakkola (1)]. They rely on simple non-verbal behaviors and the tasks can be adapted to suit the sensory and motor characteristics of different species (4, 5), thus making them ecologically valid. So far, most of the studies focusing on object permanence skills or search behavior in the different species place the emphasis on the highest level of cognitive performance in the species or the stage of cognitive performance achieved by the subjects. They normally discuss the results in comparison with other species [e.g., (4–10)], or in relation to the effects of differences in the testing procedure applied within species [e.g., (5, 6)]. This kind of goal often leads to involving test subjects which are fully mature, have ample experience with experimental settings, often with other types of cognitive tasks, and may be kept in an enriched environment compared to most of their conspecifics (e.g., experimental animals and human-raised individuals). Previous studies have aimed to trace the development of object permanence skills with longitudinal studies [for a review see (6)]. Although these studies indicate the potential cognitive skills of the species, they rarely focus on the striking individual variations shown in the different tasks (for example, variation in the level of skill at maturity or rate of skill development) and do not shed light on the causes for individual variation. According to a review by Thornton and Lukas (11), causes of individual variation in cognitive performance are, in general, understudied. Few studies of object permanence to date have taken into account the effects of prenatal stress and environmental enrichment during development (12, 13), yet prenatal, perinatal, and early postnatal environments were found to affect the cognitive development of animals (14). The direction of these effects depends on timing, length, and intensity of the stimulus, as well as the measurement applied [for review (15)]. Prenatal stress can have different effects in males than females [such as, sex tested, for a review see (16)]. For instance, male rat offspring showed impaired learning and memory skills after exposing pregnant mothers to restraint stress, while these cognitive skills were unchanged in the female offspring (17).

Recently, goat cognition has become a topic of interest as there is increasing evidence that goats can perform well in different learning and memory tasks. Goats are group-living, browsing animals and their behavior is greatly influenced by the way they perceive, process, and memorize information from their environment (18). They are able to use direct and indirect information to locate a food reward (19, 20), are capable of solving complex learning and memory tasks (21), can learn socially from humans in spatial tasks (22), and, given the opportunity, they will actually seek cognitive challenges (23). Previous work on goat cognition also shows that goats have excellent vision, responding not only to spatial and temporal variations of visual stimuli such as different shapes (24–27) but are also able to concurrently recall between five and seven different discrimination problems that they had previously learned and retained over several weeks (18). Good visual perception and learning skills can also be expected since they are prerequisites for the social recognition skills/abilities present in these animals [e.g., (18, 28); but see (29)]. These skills are crucial for goats which are a highly social species living in stable, individualized social groups (30, 31). Specific aspects of personality, namely sociability and exploration, were found to have an effect on cognitive performance in discrimination learning and non-associative food searching task in this species (32). In one study, adult dwarf goats, as a group, showed remarkable skills when a food item was hidden in one of two non-identical cups and the position of the cups were visible changed, crossing in the view of the animals (33). As goats are sensitive to aspects of their social environments, variations to their social environment such as group size, group stability, and space allocation (29, 31, 34–38) can have an effect on their cognition. For example, Langbein and colleagues found that a simple relocation (a normal husbandry routine) resulted in impaired (albeit minor) visual memory retrieval abilities in goats (39). This relationship between stress and cognitive abilities has already been well-described in human psychology and has been applied more recently (though to a lesser extent) to non-human animals (40, 41).

Adult Norwegian dairy goats are housed at relatively high animal densities during pregnancy and experience higher levels of social stress in terms of more agonistic interactions than goats kept at lower densities (38). In a parallel study, we found that prenatal social stress inflicted via high stocking densities negatively affected the behavioral development of goat kids (29). Prenatal stress is also known to affect cognition in animals during development [for reviews see (15, 42–44)]. Brain neurogenesis, structure, and function can be dramatically affected by the environmental conditions that an animal experiences during prenatal development (45–47). Specifically, the hippocampus has been comprehensively shown to be deleteriously affected by prenatal stress (48–53). Since the hippocampus processes learning, memory, and spatial and contextual information, it is probably the most crucial brain region in object permanence comprehension (54). Direct links between object permanence performance and prenatal stress (12, 13), frontal lobe activity (55, 56), and hair cortisol levels [an indicator of chronic stress; (57)] have been found. Interestingly, a parallel study conducted on sheep found a significantly higher total spine density in apical dendrites of the CA1 pyramidal neurons in the hippocampus of lambs born to mothers held at a treatment density of 1.0 m^2^ throughout gestation than lambs from 3.0 m^2^ (58). Therefore, it is likely that prenatal stress due to reduced space allowance will affect the cognitive processes of 6-week-old goat kids.

The goals of the present study were: (a) to assess the individual variation in cognitive capabilities of 6-week-old goat kids using tasks based on methods from early stages of Piaget's object permanence tasks; and (b) to examine whether prenatal stress via increased animal densities, sex of the subjects, or litter size impacted these abilities. We predicted that a high prenatal density would have negative effects on the cognitive skills of the kids and, as a result, kids born from the high prenatal density treatment would be less capable of comprehending searching tasks than kids born from the lower densities at this age. No effects of sex of the kids were predicted based on earlier studies comparing cognitive skills in goat (21).

## Materials and Methods

### Animals and Treatment During Gestation

Healthy, pregnant, dehorned Norwegian dairy goats from the experimental goat herd of the Norwegian University of Life Sciences, Ås, Norway were used in the experiment. Ethical rules stated by Forsøksdyrutvalget (the Norwegian Committee for Research Animals, www.fdu.no) which satisfy the European Union (EU) animal testing directive (86/609/EEC), the Council of Europe Convention on Laboratory Animals (ETS 123; http://conventions.coe.int/Treaty/en/Treaties/Html/123.htm) and the legislations for keeping farm animals and small ruminants in Norway (www.mattilsynet.no) were followed. In addition, all study practices were reviewed and approved by the Norwegian University of Life Sciences' Institutional Animal Care and Use Committee, The Animal Production Experimental Center.

The herd is kept on pasture in the mountains during the summer period. In September (2011), the goats were transported from pasture to the farm in Ås and were housed individually due to measurement of feed consumption in a nutritional experiment, with visual, olfactory, and limited physical access to each other, causing minimal stress in relation to isolation. Beginning in mid-October, the goats were placed into groups of 15–35. During this time, the hay and concentrate provided was reduced in order to terminate lactation. Approximately 2 weeks later, in early November, the prenatal density treatment began. The goats were not synchronized and were inseminated or mated between the end of October and mid-November. One buck was used for mating and semen from three other bucks was used for insemination. Fifty-four multiparous female goats, aged 2.8 ± 0.1 years and weighing 50.2 ± 1.0 kg were selected based on confirmation of pregnancy (by not returning to estrus and/or ultrasound investigation 3–7 weeks after mating or insemination) and expected time of parturition. These goats were evenly distributed in herds of six animals (a total of 18 animals per treatment) in densities of 1.0, 2.0, or 3.0 m^2^ per animal (low-density: pens 276 × 650 cm each; medium-density: pens 189 × 632 cm, 224 × 540 cm, 276 × 435 cm; high-density: pens 189 × 317 cm, 224 × 270 cm, 224 × 270 cm, see (38) for specifics on goat allocation and the pen densities chosen). The goats were kept in stable groups and not mixed with new individuals throughout their entire pregnancy until their kids were 5 weeks old.

The treatment pens were indoors, in one of two insulated, mechanically ventilated rooms in the same building with a constant room temperature of approximately 10°C. Artificial lighting provided a 7:17 h light: dark regime with lights on at 8 a.m. in addition to natural lighting through windows along either side of the building. The pens were made of 1.5 m high solid walls (15 mm plywood) which prevented physical contact between groups. Flooring consisted of expanded metal flooring with a 60 cm solid wood area at the rear end of the pen where sawdust was laid for bedding. The pens were cleaned in the morning and afternoon after feeding. During this time, fresh bedding was added to the solid floor area. Free access to fresh water, grass silage, and salt blocks with copper were provided. The front of each pen had six eating places (one for each goat) which provided access to a common feeding trough. Silage was supplied every morning and afternoon. The goats were also fed 0.2 kg of concentrate feed every morning throughout most of the experimental period. The concentrate was gradually increased to 0.5 kg in the last part of pregnancy (from mid-January until kidding) when the feed was complemented with hay in the afternoon to stimulate the goats' digestion. At the time of expected birth, each goat was isolated from the herd until 24 h after parturition to allow for maternal care and bonding. After the 24-h post-parturition period, the goats and their kids were returned to their treatment herd. The feed openings (eating places) in the pens allowed kids to move freely between their home pen and separate kid areas which had solid wooden floors and free access to hay. The birth of the kids was staggered over a 5-week period from the beginning of February to the beginning of March.

One goat from the medium-density treatment aborted 16 days before the expected date of parturition. This goat was removed from the experimental pen for 8 days for observation, medicated, and returned to the same experimental pen until the end of the treatment. A stillborn kid was born in the medium-density treatment (most likely due to complications at birth) and the mother could not be saved. One goat from the low-density treatment gave birth to two live and two stillborn kids (the latter two were immature). Finally, a live-born singleton kid from the high-density treatment had to be removed for a parallel study. Only data from the remaining 51 litters (low-density: *n* = 18; medium-density: *n* = 16; and high-density: *n* = 17) are presented.

### Goat Kids

**Figure 1:**
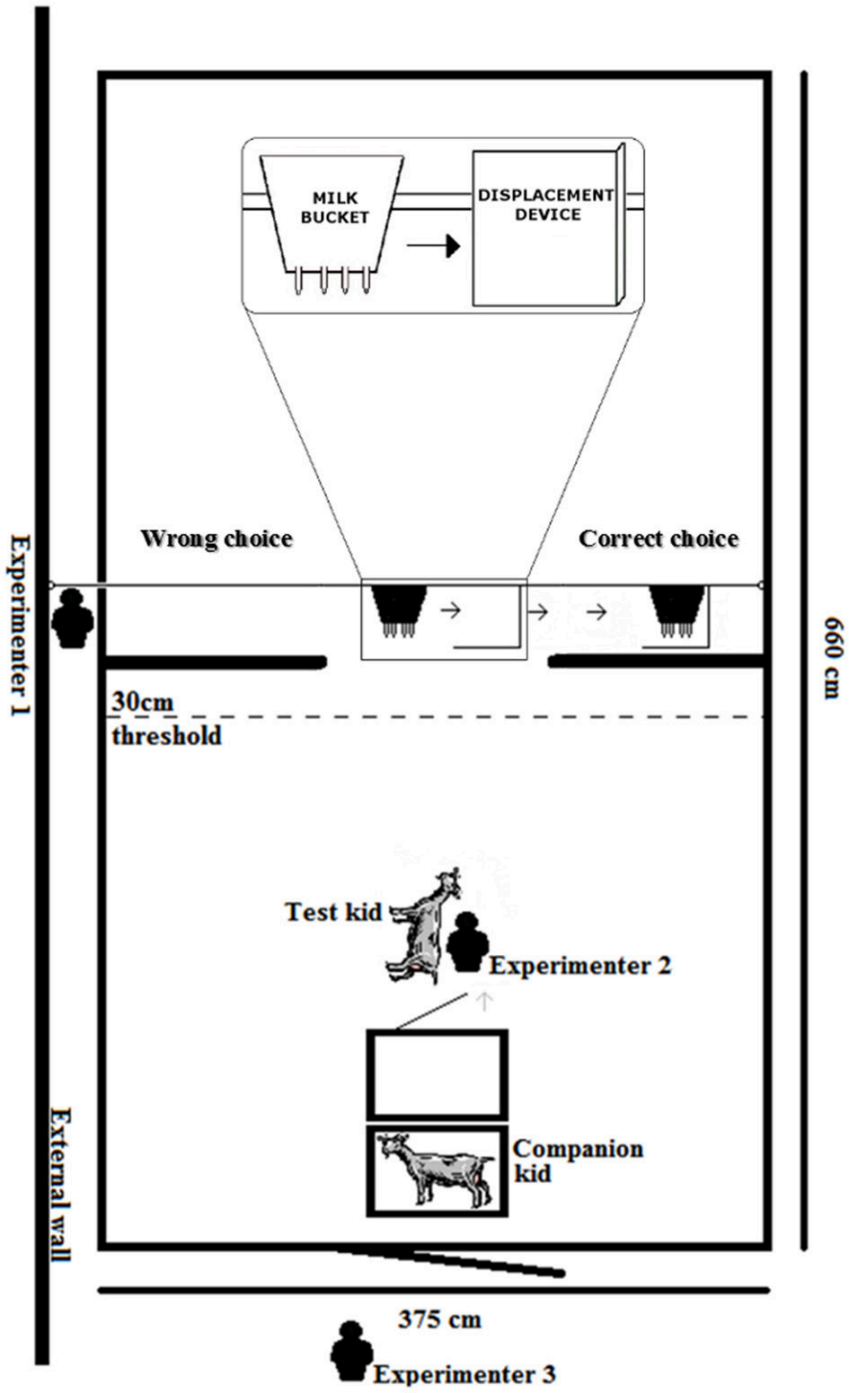
The experimental set up of the test arena. The test during the Invisible displacement task with three experimenters, the test kid, a companion kid remaining in its cage and the milk bucket sliding first into the L-shaped box then both moving behind the right screen. A choice to the left would be considered a fail while a choice to the right would be considered correct.

Beginning when the kids turned 3 weeks of age, in addition to having free access to their mothers, the kids were introduced to free access to warm goat milk from a milk bucket with four artificial teats affixed to the wall in each kid area. Each kid was also handled and hand fed via a bottle affixed with an artificial teat at least once a day. This was done to teach the kids to suckle milk from a source other than their mothers and to ensure that the kids had learned that the milk bucket was a positive stimulus. By 4.5 weeks of age, the kids' access to their mothers was blocked as per standard procedure in order to begin the weaning process and following behavioral tests at 5 weeks of age for another study investigating anxiety in a novel environment and sociality (29), the testing arena became the kids' home pen (375 ^*^ 660 cm; Figure 1). This change was carried out prior to testing to ensure that fear or stress of a novel area did not have an effect on the cognitive performance of animals (59). At this time, the kids from all treatments were housed together in this pen and the experimenters did not have access to information about their treatments. The kids had free access to the milk buckets throughout this period until the end of testing at 7 weeks of age. Milk was not provided after 17:00 the days preceding test days but free access to water and solid food (hay, silage, and concentrate) was. Prior to testing, the test kids were herded out of the test arena to a pen in a room adjacent to the experimental room to minimize pre-test handling.

### Data Collection

#### Birth Weight and Cortisol Levels

Individual kids were sexed and ear-tagged within 12–36 h after birth. At the same time, their weights were measured on an electric scale.

Blood was taken and processed as described in Vas et al. (38). Blood samples were collected from the mothers of the kids via venipuncture in the jugular vein three times during pregnancy (in the first, second, and last third of pregnancy), on two consecutive days in each period before the morning feeding (between 7:00 and 8:30). Blood samples from the kids were collected when the kids turned 3 weeks of age, on two consecutive days. All the sample collections were performed with minimal disturbance of the goats, gentle handling, and by trained and experienced assistants. Samples were collected into heparinized tubes (Vacutainer, Becton and Dickinson, Leuven, Belgium). After storage at −4°C for 2 days, samples were centrifuged at 3,000 × g for 15 min. After removing the plasma, samples were stored again at −20°C. The samples were analyzed for cortisol by the Hormon Laboratory of the Oslo University Hospital, using electrochemiluminescence immunoassay (ECLIA, Roche Cobas Cortisol assay) by using Roche Elecsys E immunoanalyzer system (Roche Diagnostics, Mannheim, Germany). For more details see Vas et al. (38). The cortisol values of the six samples from the mother goats and the two values from the kids were averaged resulting in one single cortisol value for each goat.

#### The “Search Test”

The week the kids turned 6 weeks of age, they were presented with a series of “object permanence tests” which were a modification of the tests used by Gagnon and Doré (60). The testing period was staggered over a 5-week period as there were 5 weeks separating the first birth from the last. The kids were separated into five groups accordingly.

##### Apparatus

**Figure 2:**
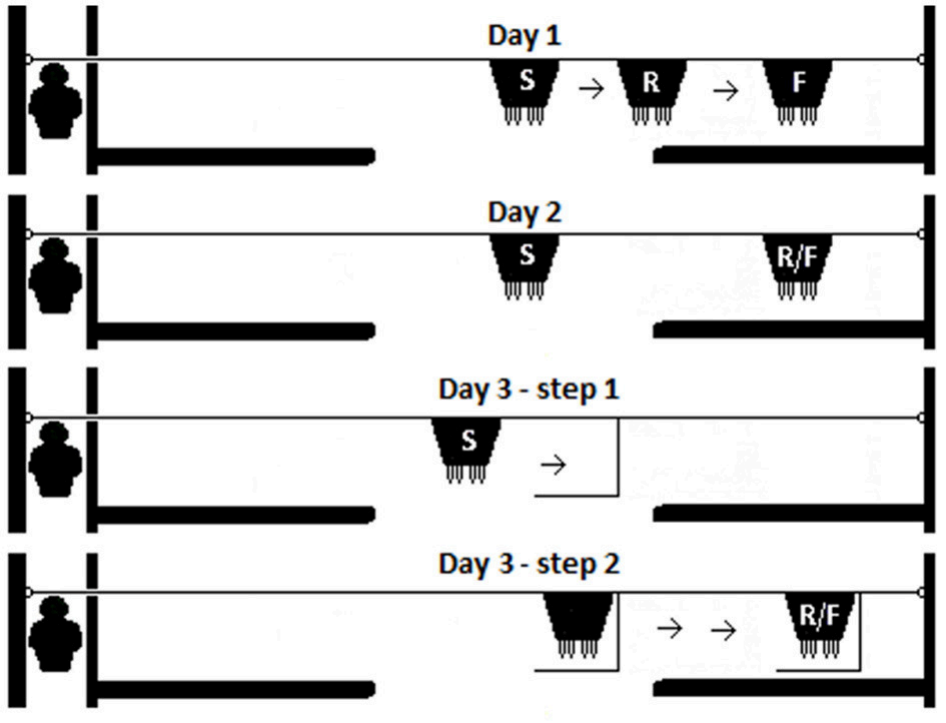
The test procedure for each day. Visible displacement with early search was tested on Day 1. On Day 2, kids were tested for their performance at the Visible displacement task. On the final day of testing (Day 3), a simple Invisible displacement task was conducted in two steps (Day 3—step 1 and Day 3—step 2). The start of each trial (S), the release of the test kid (R), and in the final position (F) of the bucket are indicated for each stage tested.

The design of the apparatus was adapted to the morphological characteristics of goat kids (60, 61). An artificial milk bucket (27 ^*^ 30 ^*^ 20 cm), identical in appearance with the one the kids were familiar with, was used as the target object. Two 125 ^*^ 114 cm opaque curtains (hereafter referred to as screens) were hung across the test arena (332 cm from the entrance door and 328 cm from the back wall; Figure 1). These provided hiding locations on either side for the milk bucket but with a 125 cm opening so that the kids had full view and access to the bucket when the bucket was positioned in the center. Forty-five centimeters behind the screens, metal wiring was strung in a loop (65 cm above the floor) with pulleys on either side allowing the milk bucket to be hung and drawn to either side behind the screens (Figure 1). The metal wire holding the milk bucket went through a hole in the wall of the test arena allowing a researcher to pull the bucket to the left or right, manipulating the movement of the milk bucket, while remaining hidden. This controlled for any inadvertent experimenter cues given through the manipulation of the bucket (such as choose the last or first box touched by either the experimenter or displacement device; see (5) for a review). The bottom of the milk bucket was approximately 30 cm from the floor, roughly the height of a goat udder. For the invisible displacement task, a 36 ^*^ 38 ^*^ 24 cm L-shaped wooden frame, which was completely covered with the same opaque fabric as the curtains, was attached on a third line so that the bucket would move behind the frame, catch the “L” and drag the frame to either side (Figures 1, 2). From the kids' perspectives, it appeared as though the milk bucket completely disappeared behind or into the displacement device. The L-shaped wooden frame (the displacer) could be attached from the top or flipped and attached by the bottom, effectively switching which side it would be dragged. As the apparatus was stored in the same building as the goats and both screens had milk splashed on them, olfactory cues were not a concern (1, 5, 60, 62–64).

##### “Habituation” trials

Before the first day of testing, habituation trials were conducted to allow the kids to become familiarized with the apparatus (10, 63). The habituation trials were done in pairs as goats are highly sensitive to separation (65–67) and a companion allowed the kids to feel more secure while becoming accustomed to the experimental set up. Competition between the two kids to gain access to the milk should not have been an issue as the milk bucket had four artificial teats and four kids were often seen sucking simultaneously before testing. Kids were also never observed “forfeiting” a teat to another kid; therefore, the most dominant kids did not monopolize the bucket during the habituation trials. The milk bucket was placed in the center of the room, equal distance from either screen with the artificial teats facing toward the cages so that the test kid had full view of both the bucket and the teats facing the kid. During the habituation trials, the cages were placed side by side parallel to the apparatus and the kids were placed in the cages. Both kids were released simultaneously and allowed 2 min from release to suck from the bucket. They were then collected, returned to the cages for 30 s and re-released. Three habituation trials were conducted in a row. If a kid latched onto the artificial teat and attempted to suck milk, they were marked as sucking. As Pepperberg (5) stated, in order for Piagetian tasks to accomplish the goal of determining levels of cognitive processing, the animal must be motivated to engage in the task at hand. Cognitive performances can be highly influenced by motivations such as the subjective value of the reward (68), being distracted by other events, and current stress level of the animal. The habituation trials were used to allow the most motivated kid to be chosen from twin litters as the test kid and exclude the non-motivated kids from further testing. Twenty-five kids were exposed to habituation trials from the low-density treatment, of which, 14 sucked. In the medium-density, 11 out of the 28 kids sucked, and in the high-density, 14 of the 22 kids sucked. If both kids from a twin litter were marked as sucking for the same amount of trial, then one kid was chosen at random to be the test kid of that litter. Twenty-six kids from the 51 litters were used as test kids (for details about number of males and females, singleton and twin kids see **Table 2**).

##### Procedure

In the test arena, two cages were placed side by side perpendicularly to the apparatus to house the test kid (in the cage closer to the apparatus) and a familiar “companion” (not a test kid; Figure 1). A “companion kid,” that was housed together with the test kid, was used to avoid the test kid performing behaviors as a result of separation from group mates (59). The “companion kid” was changed after approximately three kids were tested.

Three experimenters were used to administer the search tests (Figure 1). Experimenter 1 sat outside the test arena. Experimenter 1 was able to pull the metal wire holding the milk bucket to the left or right while remaining hidden. Half of the trials were conducted pulling the bucket behind the left screen and half behind the right in a semi-randomized order to control for side preference (for example, R-L-L-R-L-R-R-L-R-L; see Table 1 for the number of trials conducted at each stage). The pattern of the trials was alternated with the first trial beginning to the right or to the left for every other kid for all stages. Experimenter 1 sat in this spot prior to the test kid entering the room with a list stating the predetermined order of which side the bucket was to be pulled. This allowed Experimenter 1 to remain hidden until the last trial was completed and the test kid was taken out of the test room. Therefore, the test kid should not have been influenced by the presence of Experimenter 1 and Experimenter 1 had no knowledge of which kid was being tested. Experimenter 2 was responsible for handling the kid. She placed the kid in the cage, removed, and restrained the kid by gently holding its body with the head oriented toward the apparatus but preventing it from moving toward the apparatus before the predetermined release time. After the kid's release, Experimenter 2 stood motionless with her eyes averted from the test kid and the apparatus until a choice was made, she then retreated to the corner so that the kid was neither attracted to nor received any inadvertent behavioral cues from her. Upon release, the test kid was allowed 30 s to make a choice. Once the kid passed the 30 cm threshold before the screens (Figure 1), a choice was considered to have been made. The test kid had to walk behind the correct screen to access the bucket. If the kid sucked on the milk bucket within the 30 s of the trial, it was allowed to suck for 10 s regardless of whether the choice was considered a successful choice or not. This allowed for reinforcement of the stimulus, prevented any behaviors of frustration which may have arisen upon not receiving an expected milk reward, and, most importantly, prevented any potential negative feedback of the procedure where each failure to find the object behind a selected screen could result in an extinction trial for the association. The kid was collected and placed in the cage after it had sucked 10 s or until 30 s had past if the kid did not suck, whichever occurred first. The test kid spent 30 s in its cage in between each trial. Experimenter 2 manipulated a stopwatch timing the trials, collected the kid, and placed it in the cage between trials. Experimenter 3 stood outside the test arena and recorded whether a kid was successful on each trial (see “Scoring” for passing criterion).

**Table 1:** Passing criterion for each stage tested.

**Stage tested (test day)**	**Number of trials conducted**	**Passing criterion (number of correct choices)**	**Probability^a^**	**Probability of reaching stage^b^**
VDE^c^ (Day 1)	10	8	0.05	0.05
VD^d^ (Day 2)	11	8	0.11	< 0.001
IVD^e^ (Day 3)	10	8	0.05	< 0.0001

a *Probability of reaching success criterion at current stage by chance*.

b *Probability of a single kid advancing through the previous stages and reaching success criterion at current stage by chance*.

c *The Visible displacement with early search task*.

d *The Visible displacement task*.

e *The Invisible displacement task*.

Four test scenarios were administered over three consecutive days within the same week with the stages increasing in difficulty each day [Figure 2; (4, 61, 63)]. Three tests were visible displacement problems (Days 1 and 2) and one was invisible displacement (Day 3). On all days, the following pre-test procedure was conducted: The cages were placed adjacent to the apparatus at the entrance of the experimental pen. The milk bucket was placed in the center of the room filled with milk equidistant from either screen. The “companion kid” was brought into the test room, allowed to roam freely in the test arena, and suck from the milk bucket for 2 min and then placed in the cage. This was done in order to facilitate calm behavior of the “companion kid.” Afterwards, the test kid was brought into the test room and placed into the adjacent cage. Each test kid (in the cage closest to the apparatus (Figure 1) was tested individually. The test kid was gently removed from the cage, held facing the apparatus directly in front of the cage, and released without moving the bucket to allow the kid to suck on the bucket as a warm-up trial. After 30 s, the test kid was collected and put into the cage. This pre-test warm-up trial was conducted to reinforce the milk bucket as a stimulus.

*Single visible displacement with initiation of the search movement (“Visible displacement with early search”)* When 60 s had passed after the kid was placed in the cage after the initial warm-up trial, Experimenter 2 removed the kid from the cage and held it as during the warm-up trial. The bucket was slid horizontally and the test kid was released *as* the bucket began to go behind the screen (Figure 2: Day 1).

*Single visible displacement (“Visible displacement”)* If the Visible displacement with early search stage permanence criteria was achieved (see later criteria), the kids were tested in Single visible displacement tasks. The testing procedure was identical to Day 1 except that the bucket was slid horizontally until fully concealed *before* releasing the test kid. The kid was released after all movement of the bucket was completed (Figure 2: Day 2). The pattern was changed and an 11th trial was added which allowed for three consecutive trials to be conducted behind the same screen (for example, R-L-L-R-L-R-R-L-L-L-R). Care was taken that the number of consecutive trials to one side was less than five to control for trial and error/place learning (69, 70). Again, the pattern of the trials was alternated for every other kid.

*Simple invisible displacement task (“Invisible displacement”)* Based on traditional invisible displacement tasks and invisible transposition tasks administered to dogs (*Canis familiaris:* (7, 71), cats (*Felis catus:* (71), and jackdaws (*Corvus monedula:* (72) the milk bucket was slid horizontally into a displacement device (the L-shaped box) so that it was completely hidden in full view of the test kid (Figure 2: Day 3—step 1). The bucket remained in the displacement device while it (with bucket behind) was slid, in full view of the kid, behind either test screen (Figure 2: Day 3—Step 2). The kid was released when both the bucket and the displacement device had come to a complete stop.

*Scoring* Kids were deemed successful on a trial if the orientation of their head was toward the side the bucket was positioned when it was at the threshold 30 cm before the screen (Figure 1) and sucked from the milk bucket. A threshold of 30 cm before the screen was chosen as that was the last point where the test kid could not see the bucket behind either screen if they were positioned in the center as they approached the screen. If a kid suddenly looked the opposite way of its trajectory (as if to check behind the other screen) and/or suddenly changed trajectory from the wrong side to the correct side after the 30 cm threshold, it was recorded as an incorrect choice as it was assumed the kid simply saw the bucket. Similar to other studies, a response was scored as incorrect when the kid chose the wrong screen. A trial was scored as “no choice” when the kid did not make a response within 30 s of release and the trial was not included in the total number of trials given for analysis. Although it may be that “no choice” was made due to lack of motivation or because of uncertainty about the location of the reward, these two causes could not be distinguished behaviorally. In total, there were only 8 trials where kids did not make a choice. Two portable cameras (SONY HDR-SR12) were set up at either side of the test arena to record behaviors. In addition to recording behaviors through direct observations, choices were confirmed via video analyses by one experimenter. In the case of discrepancies recorded through direct observations and video analyses (0.4% of trials fell under this category), a minimum of two experimenters reanalyzed the footage. All experimenters were blind to the treatment condition of each kid.

The number of trials conducted at any stage were kept to a minimum to control for the possibility of training or learning [see e.g., (5, 73)] and to avoid saturation. Ten trials were conducted each day with the exception of the extra trial on Day 2, when eleven trials were given. In all conditions when a choice was made the probability of chance success was 0.5. Therefore, according to the exact binomial tests (57, 60, 74), passing criterion was as in Table 1. Consequently, subjects were failed and testing was discontinued if three trials were scored as “incorrect” in total at a single stage. Since the search tasks were administered with the stages increasing in difficulty (4, 61, 63) it was unlikely that if a kid failed a stage it would advance to succeed at the next, more difficult stage (60, 70); therefore, if a kid failed at a stage then the test was terminated for the kid.

Two kids which had successfully performed at the “Visible displacement task” were not tested on the “Invisible displacement.” Direct observations had marked them as failing at the visible displacement; however, upon later analyses of the videos, it was determined that they had, in fact, successfully completed that stage. Overall, 664 trials were included in analyses (not including warm-up trials), 567 of which were scored as “successful.”

### Statistical Method

R statistics software (Version 3.3.3) was employed to run all statistical models. Birth weight, maternal and kid cortisol values were standardized by twice of the standard deviation to deal with skewed distribution (the mean was subtracted from the value and then, divided by twice of the standard deviation of the sample).

First, effect of sex and litter size on birth weight were tested with Kruskal-Wallis tests. Generalized linear models were applied (with binomial distribution, log link) to evaluate the effect of sex (female or male kid) and litter size (singleton or twin) on the number of successful choices compared to the total number of trials when the kid made a choice. In addition, the treatment (with three levels: low, medium, or high prenatal density), standardized values of the following, continuous variables: blood cortisol level of the mothers, that of the kids, and the birth weight of the kids were added as covariates to the models.

## Results

Males had higher birth weights compared to females (Chi^2^ = 7.172, df = 1, *P* = 0.007; mean ± SE for males: 3.69 ± 0.14 kg, females: 3.00 ± 0.20 kg). Kids born as singletons or twins had comparable weights at birth (Chi^2^ = 1.123, *df* = 1, *P* = 0.289, singletons: 3.59 ± 0.24 kg. twins: 3.39 ± 0.15 kg).

As an overview Table 2 presents the success rate (number of successful choices divided by number of trials when a choice was made) of the kids in the different treatment groups, sexes, and litter sizes at the three stages together with sample sizes.

**Table 2:** Sample sizes, means and interquartile range (IQR) of success rates at different stages.

		**Sum**	**Prenatal maternal density^a^**			**Sex**		**Litter size**	
			**High**	**Medium**	**Low**	**Females**	**Males**	**Singleton**	**Twin**
Visible early	N^b^	26	10	7	9	9	17	8	18
	Mean^c^	0.915	0.940	0.914	0.887	0.864	0.941	0.988	0.882
	Lower quartile	0.900	0.925	0.850	0.900	0.800	0.900	0.900	0.825
	Higher quartile	1.000	1.000	1.000	1.000	1.000	1.000	1.000	1.000
Visible	N^b^	22	8	7	8	7	16	8	15
	Mean^c^	0.800	0.854	0.721	0.815	0.846	0.780	0.831	0.783
	Lower quartile	0.730	0.798	0.640	0.745	0.820	0.708	0.730	0.685
	Higher quartile	0.910	0.910	0.820	0.933	0.910	0.910	0.910	0.910
Invisible	N^b^	16	6	4	6	6	10	5	11
	Mean^c^	0.829	0.800	0.875	0.828	0.783	0.857	0.900	0.797
	Lower quartile	0.800	0.800	0.825	0.800	0.800	0.800	0.800	0.700
	Higher quartile	1000.000	0.875	1.000	0.950	0.800	1.000	1.000	1.000

a *Space allowance of 1.0 (High), 2.0 (Medium), or 3.0 (Low) m^2^ per animal provided to pregnant goat mothers*.

b *Number of subjects participating*.

c *Mean of the success rate, which is successful choices divided by number of trials when a choice was made*.

### Visible Displacement With Early Search

**Figure 3:**
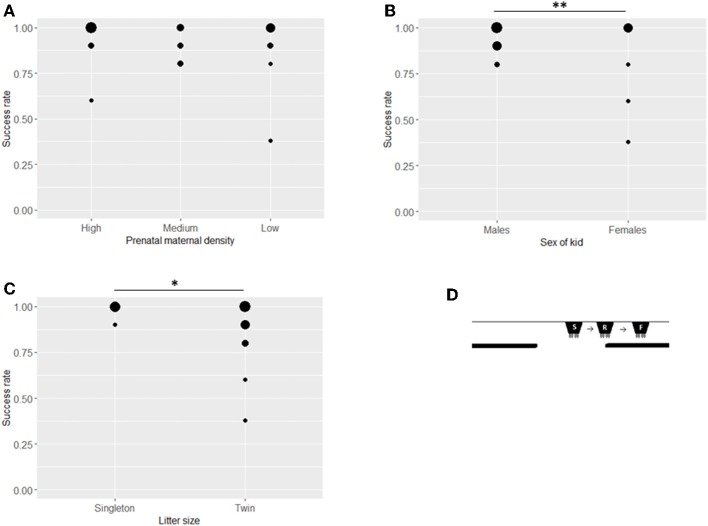
Success rates of kids in the Visible early task. Success rates (number of successful choices divided by number of trials when a choice was made) in the Visible early task by treatment **(A)**, sex **(B)**, and litter size **(C)**. The position of the bucket at the start of each trial (S), the release of the test kid (R) and in the final position (F) in the Visible early task **(D)**. Asterisks indicate significant differences between groups (at ^*^P < 0.05 and ^**^P < 0.005 level). Size of dots refer to number of overlapping data points.

In this first stage (Figure 3D), 26 goat kids participated, with an average success rate of 91.5% (Table 2). Prenatal maternal density had no significant effect on the success rate of early search of visible displacement of the kids (ß = 0.016, *SE* = 0.328, *z* = 0.049, *P* = 0.960, Figure 3A) but males performed better than females at this stage (ß = −1.985, *SE* = 0.665, z = −2.985, *P* = 0.002, Table 2, Figure 3B) and singleton kids had a higher success rate than kids from twin litters (ß = −2.429, SE = 1.082, *z* = −2.245, *P* = 0.025, Table 2, Figure 3C). Neither the cortisol level of the mother (26.06 ± 1.81 nmol/l, ß = 0.198, *SE* = 0.289, *z* = 0.685, *P* = 0.494) nor that of the kid (29.52 ± 7.15 nmol/l, ß = 0.016, *SE* = 0.247, *z* = 0.064, *P* = 0.949) affected the success rate significantly but kids with higher birth weights had lower success rates (ß = −1.218, SE = 0.388, *z* = −3.140, *P* = 0.002).

### Visible Displacement

**Figure 4:**
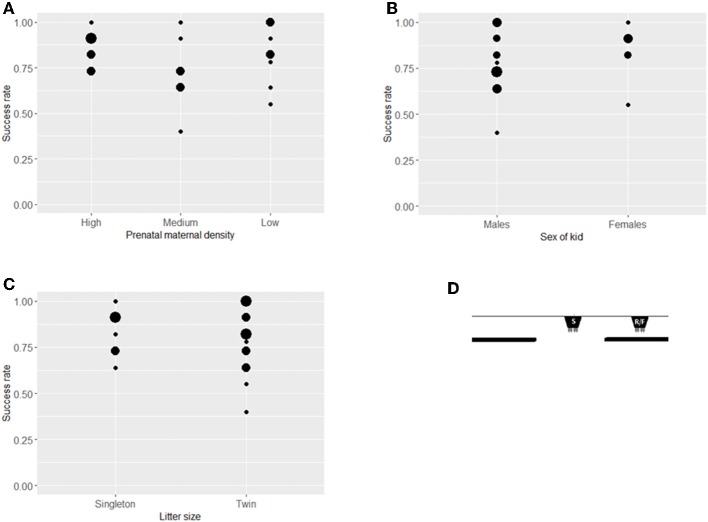
Success rates of kids in the Visible displacement task. Success rates (number of successful choices divided by number of trials when a choice was made) in the Visible displacement task (mean ± SE) by treatment **(A)**, sex **(B)**, and litter size **(C)**. The position of the bucket at the start of each trial (S), the release of the test kid (R) and in the final position (F) in the Visible displacement task **(D)**. There was no difference between groups at *P* < 0.05 level. Size of dots refer to number of overlapping data points.

In sum, twenty-three kids were tested in the visible displacement task (Figure 4D). Prenatal maternal density had no significant effect on success rate in the visible displacement task (ß = −0.146, *SE* = 0.216, *z* = −0.675, *P* = 0.450, Figure 4A). The two sexes and kids from singleton vs. twin litters showed a similar performance (sex: ß = −0.058, *SE* = 0.546, *z* = −0.106, *P* = 0.915, litter size: ß = −0.508, *SE* = 0.372, *z* = −1.365, *P* = 0.172; Figures 4B,C). Maternal and kid cortisol levels and birth weight did not affect the performance (maternal cortisol: ß = 0.319, *SE* = 0.209, *z* = 1.526, *P* = 0.127, kid cortisol: ß = −0.043, *SE* = 0.176, *z* = −0.244, *P* = 0.808, weight: ß = −0.263, *SE* = 0.223, *z* = −1.178, *P* = 0.239).

### Invisible Displacement

**Figure 5:**
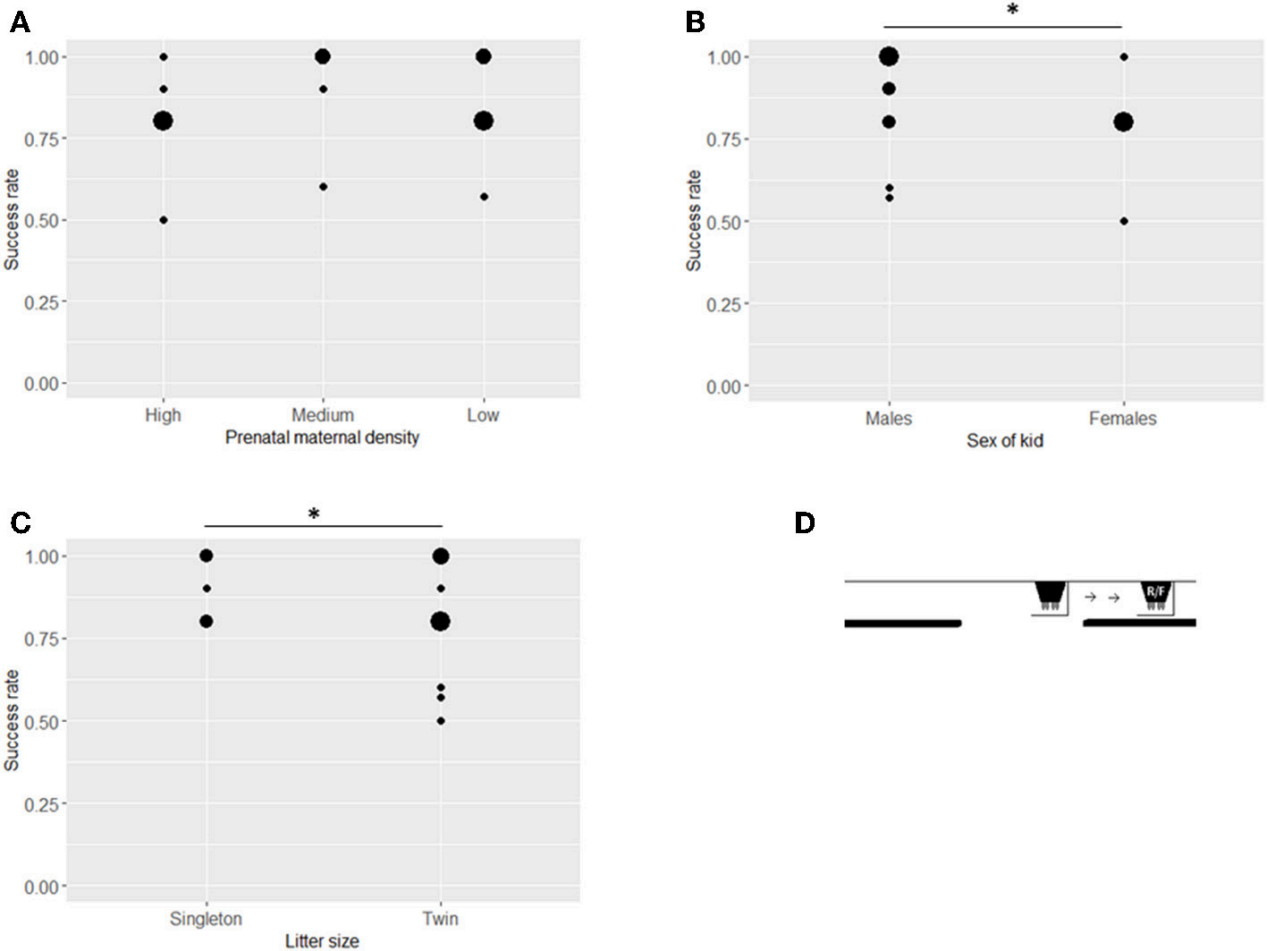
Success rates of kids in the Invisible displacement task. Success rates (number of successful choices divided by number of trials when a choice was made) in the Invisible displacement task (mean ± SE) by treatment **(A)**, sex **(B)**, and litter size **(C)**. The position of the bucket at the start of each trial (S), the release of the test kid (R) and in the final position (F) in the Invisible displacement task **(D)**. Asterisks indicate significant differences between groups (at *P* < 0.05 level). Size of dots refer to number of overlapping data points.

Sixteen kids participated in Invisible displacement tasks (Figure 5D). There was no effect of prenatal maternal density on performance (ß = −0.514, *SE* = 0.378, *z* = −1.359, *P* = 0.174, Table 2, Figure 5A). Males had a higher success rate compared to females (ß = −1.736, *SE* = 0.766, *z* = −2.265, *P* = 0.024, Table 2, Figure 5B) and singleton kids performed better than kids from twin litters (ß = −1.110, *SE* = 0.566, *z* = −1.961, *P* = 0.050, Table 2, Figure 5C). A higher level of maternal cortisol level was associated with a higher success rate at this stage (ß = 0.677, *SE* = 0.325, *z* = 2.082, *P* = 0.037). Kid cortisol level or birth weight did not have any significant effect on success rate in the invisible displacement task (kid cortisol: ß = 0.321, *SE* = 0.235, *z* = 1.363, *P* = 0.173, birth weight: ß = −0.202, *SE* = 0.338, *z* = −0.597, *P* = 0.550).

## Discussion

The adult goats (mothers of the test kids in the present study) kept in the high density showed more agonistic (offensive and defensive) and slightly less socio-positive interactions compared to goats kept in the low density in a previous study (38). Interestingly, blood cortisol levels of the mothers were found to be comparable in the different density treatments in our earlier study (38), possibly indicating individual variation in line with individual responses to the varying space allowance or other factors. Kids of these mothers were the subjects of the present study. However, contrary to what was predicted, the applied prenatal density treatment as an expected cause of prenatal stress did not affect the performance of the kids at any level of the cognitive tasks.

Although maternal space allowance, *per se*, had no effect, kids whose mothers had higher cortisol values during pregnancy performed better in the Invisible displacement search task. Goat kids were subjected to three different tasks. In the Visible displacement task with early search, kids could initiate searching behavior when the target object was only partly hidden and partly seen. At this stage, simply following the trajectory of the object is enough to be successful and no memory is required. At the next stage, during the Visible displacement task, kids were restrained for a longer time and could begin to approach only when the target object was no longer visible. In this task, although some level of working memory is required, subjects approaching the place where the target disappeared could easily find the target. In the Invisible displacement task, however, the target disappeared at first behind an occluding barrier and moved further in occlusion to its final destination. To be successful in this task, kids had to follow the trajectory of the movement further compared to where the target disappeared from view. This stage is regarded as the most cognitively demanding in our setting because of the longer time gap between the last point at which the target is seen and the initiation of movement as well as the longer distance between the last point at which the target is visible and its final location.

There are several possible factors which can, to some degree, explain individual variations in cognitive skills. Prenatal stress, stress experienced by pregnant mother during pregnancy, and its effect on offspring are understudied and the results, mainly in rats, are complex. Different kinds of prenatal stress were found to increase [e.g., (74)] or, in the majority of studies, decrease cognitive performance in the offspring [e.g., (49, 51, 75–78)]. The significance of the effect also depends on the type of cognitive skill and the specific methods applied to evaluate it (79). An intense, long-lasting prenatal stress in the period of pregnancy when the HPA axis is developing (timing depending on species) is hypothesized to impair cognitive development and skills in the offspring [for reviews in humans see (80–84), reviews in animals e.g., (14–16, 85)]. At the same time, prenatal maternal stress was found to facilitate development of cognitive skills in some studies. For instance, language skills were improved in prenatally stressed 11 years old girls compared to non-stressed girls (86). Improved cognitive skills were also found in children exposed to stress prenatally in another human study (87). The explanation for these contradictory effects of prenatal stress most likely lies in the timing of the stressor as mid-gestational stress was associated with improved learning in two studies on male rat offspring (88, 89).

The findings of no effect of density but a link to maternal cortisol suggest that prenatally elevated maternal cortisol levels could lead to enhanced cognitive skills in goat kids. The interpretation of blood serum cortisol level as indicator of stress has to be done with caution due to multiple factors affecting corticosteroide levels (90). Still, it is often used as indicator of acute or chronic stress in animals under experimental conditions, where experimental setup (e.g., multiple sampling) and animal management (e.g., strict feeding regimes) can control for some of the possible environmental (e.g., feeding time) and animal-related (e.g., species, breed, lactation status, age of animals) factors leading to variation in corticosteroide levels. Producing offspring with better cognitive abilities in challenging, moderately stressful, and unpredictable environments may be adaptive from an evolutionary point of view as the offspring may be more capable of handling cognitive challenges. Furthermore, the inconsistency in our results regarding treatment and maternal cortisol suggests that individual stress in pregnant goats may be indicated more directly by maternal cortisol levels than space allowance measured at group level. The applied animal densities in this study were in range of the common practice in Norway and complied with space allowance regulations of goats kept indoors within the European Union. The experimental densities were aimed to model commercial conditions and suggested that even the highest animal density represented a manageable, moderate stress level for goats. Similar managed environments may impact individuals differently based on, for instance, the group composition including behavioral profile of group members, individual relationships, the animals' rank position, and coping style at least when the environment is not too restrictive. Therefore, our findings emphasize the importance of individual animal-based welfare indicators compared to resource-based measures.

An additional cause of observable individual variations in cognitive skills may be the sex of the animals. In our study, males had higher success rates both at the low-level Visible displacement with early search and at the highest level tested in the Invisible displacement tasks compared to females, while the performance of the two sexes was comparable in the Visible displacement task. Males weighed more at birth compared to females but animals with lighter birth weight performed better in the Visible displacement with early search task. Therefore, weight cannot be a simple explanation for the found difference between the two sexes. We can speculate that the difference at least in the Visible displacement with early search task, where there was no need for high cognitive abilities, may be caused by a stronger motivation to feed in males or higher stress levels in females. Considering the better performance of males in the Invisible displacement task, several studies indicate that there are sex differences in specific aspects of cognitive skills. The presence, absence, or direction of differences are highly dependent not only on the skill but on the methods used to evaluate (77, 79). Regarding cognition in goats specifically, no sex differences were reported in adult goats learning and recalling a new object manipulation task (21) and no differences were found between males and females in visual discrimination and a non-associative cognition task in another study (32). Furthermore, female and male goat kids were equally unable to discriminate between a familiar and an unfamiliar test kid in a social discrimination task (29). This study may be the first reporting sex differences in cognitive skills in goats. Here, we raise several hypotheses for this observed sex difference, particularly in the Invisible displacement task. First, that the observed superior performance of males over females is a true difference in cognitive skills in these tasks. Most of the published goat cognition studies involve subjects from a single sex [e.g., only males (91, 92), only females (18, 19, 23, 25, 28, 93–95)] or both sexes but their performance is not compared (20, 21, 26, 96). In earlier object permanence studies, no sex differences were reported to our knowledge possibly partly because this comparison was not the focus in the majority of studies. Second, cognitive performance may be influenced by motivation (68). All subjects were exposed to habituation trials to pick the highest motivated kid from twin litters and to involve only motivated kids before the test trials. At the same time, motivation could change from day to day according to actual needs, for instance, depending on hunger, thirst, tiredness, or stimuli immediately before testing. Third, while a mild level of stress (arousal) may enhance cognitive performance, high levels of stress can impair attention span and other relevant skills (40, 41). In an earlier study, female and male goat kids of comparable age were found to have similar basal cortisol values and responded similarly in a social test (35). A social isolation and a social test performed on the subjects in the present study were reported in a separate paper (29). There, males showed lower levels of sociality (measured as approaching stimuli kids in an unfamiliar arena) but there was no difference between males and females in the number of vocalizations or escape attempts made in a social isolation situation. Fourth, cognitive impairment caused by prenatal stress can be reversed by early postnatal environment, e.g., by secure attachment between infant and mother in humans (76), better mothering skills in rodents (97), or environmental enrichment in rats (98, 99). It may be that even though males and females were exposed to a similar amount of prenatal stress and similar postnatal environments, the prenatal stress had different effect on cognitive skills in females than in males. This was shown, for instance, in passive avoidance learning of rats of mothers exposed to restraint stress during pregnancy (77).

The blood serum cortisol level of kids at 3 weeks age collected by venipuncture did not predict later performance of these kids in the search tasks. A relationship was predicted, as both blood sampling and testing procedures included handling of the test kids and some restraint. Although blood was collected by experienced assistants and the measurement was aimed to indicate basal cortisol levels, we cannot exclude that the young kids, less used to human handling reacted to the procedure and cortisol levels were raised by the actual time of sampling. In theory, it is possible that individuals more reactive to an environmental stimuli (kids with higher blood cortisol values) would be more aroused in moderately stressful situations and this increased arousal would lead to better cognitive performance or, if the situation causes a high stress reaction, cognitive performance could be impaired. By the time of testing, kids were regularly handled by the experimenters and habituation and warm-up trials were planned to eliminate unnecessary stress. Therefore, we assume that the stress reactivity of the goat kids played a minimal role in successfulness in the search tasks and this can be an explanation to the lack of relationship between blood serum cortisol values at 3 weeks of age and cognitive performance at 6 weeks of age.

Higher performance was shown in the Visible displacement with early search task by kids with lower birth weight. This task probably did not require a high level of cognitive performance as the search behavior was initiated when the target object was still visible for the kids. Therefore, the difference in the success rate might be a consequence of difference in motivation or stress level in the kids. Kids born as singleton were often found to be heavier at birth [e.g., (88, 100–102)] and at later ages, up to until 90 days old, than kids born in twin litters [e.g., (89)] but no difference was found here in birth weight between singletons and kids born as twins similarly to an earlier study (35). Furthermore, singleton kids gain more weight on a daily average compared to kids from twin litters (89, 103), but to our knowledge, there is no information about whether higher weight gain of singletons is paralleled with higher motivation to feed. Baxter et al. (88) report differences between singletons and twins in frequency of sucking attempts made, singleton kids having more contacts at the udder and more unsuccessful sucking attempts compared to twin kids. In the present study, singleton kids performed better both in the Visible displacement with early search task and in the Invisible displacement task. Presumably, singleton kids may receive more nutrients [singleton lambs are usually heavier at birth and have lower mortality rate compared to twin lambs (104)] and maternal care from their mothers, and these nutritional and social benefits can lead to enhanced cognitive development. Although ewes with twin litters show higher total maternal investment indicated by more high-pitched vocalization (indicating anxious behavior) and more grooming behavior (indicating better caretaking) early postnatally (104, 105), which may be comparable to goat mothers with singleton or twin litters, the maternal effort is less then doubled leading to lower maternal care per offspring in twin litters compared to kids born as singleton offspring. Early postnatal environment, and especially maternal care and maternal style has an important effect on behavioral development in many mammalian species and higher maternal care can facilitate stress-resilience and cognitive skills (16, 97, 104–108).

In this study, we aimed to place emphasis on individual differences in cognitive skills, namely search behavior in goat kids, and to evaluate the effects of factors which can contribute to the variation in this skill. We demonstrated variations in search behavior at different levels in young goats, a new candidate species for cognitive research. Goat kids were tested at a specific age of development: 6 weeks old. In commercial herds in Norway, kids are separated from their mothers and solid food is introduced in an increasing amount at this age. Therefore, motivation for exploration, neophilia, and memory and learning skills are crucial in coping in farm conditions.

In summary, prenatal maternal animal densities did not affect performance of 6-week-old goat kids in a search task, but elevated maternal cortisol levels during pregnancy contributed to better cognitive skills in the offspring. Males and kids from singleton litters outperformed females and kids from twin litters at higher levels of searching tasks and searched more successfully at earlier stages.

## Author Contributions

JV contributed to the planning and design of the experiment, data collection, statistical analysis, and manuscript writing. RMC contributed to planning and design of the experiment, main responsibilities lay in data collection, and manuscript writing. ILA contributed to the planning of the experiment and manuscript writing as the leader of the project. All authors have read and approved the last version of the manuscript.

### Conflict of Interest Statement

The authors declare that the research was conducted in the absence of any commercial or financial relationships that could be construed as a potential conflict of interest.
